# Improving light availability and creating high-frequency light–dark cycles in raceway ponds through vortex-induced vibrations for microalgae cultivation: a fluid dynamic study

**DOI:** 10.1007/s00449-024-03074-5

**Published:** 2024-08-12

**Authors:** Mehmet Sadik Akca, Omer Kemal Kinaci, Bulent Inanc

**Affiliations:** 1 Department of Environmental Engineering Faculty of Civil Engineering, Istanbul Technical University, Istanbul, Turkey; 2 Faculty of Naval Architecture and Ocean Engineering, Istanbul Technical University, Istanbul, Turkey; 3 Department of Mechatronics Engineering, Istanbul Technical University, Istanbul, Turkey; 4 Marine Cybernetics Advanced Vehicle Technologies (MARNETICS), Istanbul, Turkey

**Keywords:** Raceway ponds, Microalgae, Light availability, Fluid dynamics, Light–dark cycles, Vortex-induced vibrations

## Abstract

Limited light availability due to insufficient vertical mixing strongly reduces the applicability of raceway ponds (RWPs). To overcome this and create light–dark (L/D) cycles for enhanced biomass production through improved vertical mixing, vortex-induced vibration (VIV) system was implemented by the authors in a previous study to an existing pilot-scale RWP. In this study, experimental characterization of fluid dynamics for VIV-implemented RWP is carried out. Particle image velocimetry (PIV) technique is applied to visualize the flow. The extents of the vertical mixing due to VIV and the characteristics of L/D cycles were examined by tracking selected particles. Pond depth was hypothetically divided into three zones, namely dark, light Iimited and light saturated for detailed analysis of cell trajectories. It has been observed that VIV cylinder oscillation can efficiently facilitate the transfer of cells from light-limited to light-saturated zones. Among the cells that were tracked, 44% initially at dark zone entered the light-limited zone and 100% of initially at light-limited zone entered the light-saturated zone. 33% of all tracked cells experienced high-frequency L/D cycles with an average frequency of 35.69 s^−1^ and 0.49 light fraction. The impact of VIV was not discernible in the deeper sections of the pond, due to constrained oscillation amplitudes. Our findings suggest that the approximately 20% increase in biomass production reported in our previous study can be attributed to the synergistic effects of enhanced L/D cycle frequencies and improved light availability resulting from the transfer of cells from dark to light-limited zones. To further enhance the effectiveness of VIV, design improvements were developed. It was concluded that light availability could be significantly improved with the presented method for more effective use of RWPs.

## Introduction

Microalgae are regarded as promising feedstock for the production of sustainable energy and fuel, as well as food and feed supplements and fine chemicals. According to Chisti [1] with microalgae having 30% lipid content, areal lipid productivity is enhanced about 10- to 340-fold comparing energy crops such as maize, palm, and canola. This potential attracted considerable attention for microalgae-based fuel and energy production.

Raceway ponds—RWPs—are the most common microalgae cultivation system both for biomass production and wastewater treatment with microalgae. It has been reported that about 90% of commercial-scale algae production is held in RWPs [2, 3]. RWPs can be defined as an oblong channel where suspension is circulated most commonly by a paddlewheel, while flow velocities vary between 0.2 and 0.5 m/s [3] and the depth is usually kept around 0.3 m to ensure sufficient light for photosynthesis.

The main advantages of RWPs compared to closed photobioreactors—PBRs—are decreased capital and operating costs, operation simplicity and scalability [3]. However, biomass productivity in RWPs is lower compared to closed PBRs due to a number of reasons, such as limited light availability resulting from insufficient vertical mixing, vulnerability to environmental conditions and contamination by other organisms. Among these, light limitation is a main drawback of RWPs and improving light availability is the most important factor to optimize productivity. Furthermore, efficient light utilization and minimum light limitation are among the main challenges for mass culturing of microalgae [4]. Another problem about RWPs is the huge area demand due to shallow ponds which clearly limits their utilization.

Adequate mixing is essential for providing frequent light energy to cells, improved cell–nutrient contact, gas exchange and gas to liquid mass transfer [5–7]. To exemplify, unmixed lagoons at Mexico demonstrate productivity of 2 g/m^2^d [4], which is about tenfold lower than that in RWPs [8].

Vertical mixing is more important than horizontal mixing in RWPs, as it directly determines light availability for cells. Vertical mixing in RWPs can be improved in certain ways. Mendoza, et al. [3] reported that vertical mixing in RWPs is limited to sump paddlewheels and bends. Thus, introducing more bends would improve vertical mixing. However, most of the head losses in RWPs arise from bend sections, which makes it energy intensive [9]. Another method is increasing turbulence by increasing flow velocity which also increases operational costs.

The existing research literature aimed to improve light availability through the creation of vortices and the upward direction of flow. The methods developed for this purpose are mainly installing static structures to flow field to create light/dark (L/D) cycles to induce so-called flashing light effect, such as alternatively permutated conic baffles, foil wings or up–down chute baffles [5, 10–13].

While it has been possible to improve biomass productivity by the above-mentioned methods, depth was very limited in these works such as to 6–10 cm, which would cause huge area demand and increased evaporation losses. Furthermore, the paddlewheel speed in most of these studies was kept increasingly high, such as 30–50 rpm, which does not represent the conditions of full-scale RWPs.

Murphy et al. [14] reported that around 90% of net O_2_ production in RWPs is limited to the first 12–13 cm of the culture. Sutherland et al. [15] reported that up to one-third of the depth in RWPs receive insufficient light to support net photosynthesis. Furthermore, the channel length of RWPs can be very long, which means most of the biomass would reside in regions of ponds for a prolonged period where there is not enough light energy to photosynthesize. On the other hand, in their investigation into the characteristics of L/D cycles occurring in RWPs, Chen et al. [16] designated the initial 3 cm of a 30 cm-deep RWP as the light zone, with the remaining portion classified as the dark zone. Assuming some parts of the reactor as “light” and “dark” have drawbacks and creates confusion to fully evaluate the impact of light experienced by cells in real reactors, as cells are indeed exposed to a light gradient rather than direct light on/off pattern through pond depth. Thus, in this study, in contrast to the existing literature, the categorization of pond depth was expanded into three zones, diverging from the conventional two.

Since limited light availability resulting from flow behavior of RWPs significantly restricts their biomass production capacities, it is essential to develop new energy-efficient methods to enhance vertical mixing. Vortex-induced vibrations (VIV) represent a phenomenon where a body undergoes dynamic motion by harnessing the hydrokinetic energy of the flow, thereby eliminating the need for any additional energy input. By installing a VIV system in an RWP, vertical flow zones can be created through continuous cylinder oscillation. In our previous work [17], we observed approximately a 20% increase in biomass production with the use of a VIV system. However, a better understanding of effect of VIV on biomass production and possible design modifications require a more detailed analysis of flow in the pond. In this study, the flow in the RWP was visualized with particle image velocimetry (PIV) technique and cell trajectories affected by the VIV system were obtained. By analyzing cell trajectories, it is aimed to understand and quantify the extent of improvement in vertical mixing achieved by VIVs.

## Materials and methods

### Pilot-scale RWP retrofitted with the VIV system

**Fig. 1:**
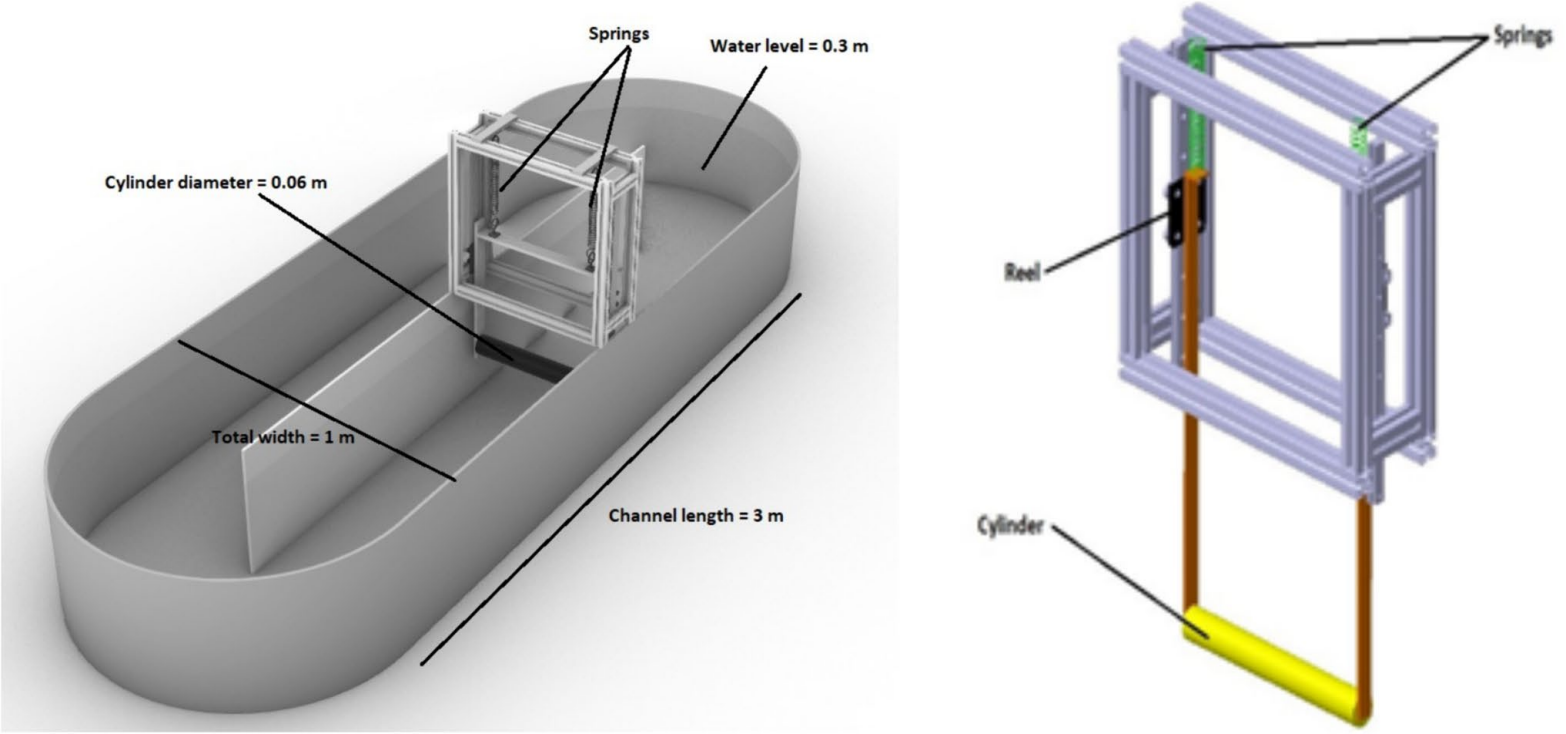
CAD drawing of the VIV system implemented to the RWP channel (left) and details of the VIV system components (right) [17]

Pilot-scale RWP has 3 m channel length and 1 m total width as shown in Fig. 1 (left). Water is circulated by a ten-blade paddlewheel with the help of a 2.2 kW AC motor. The paddlewheel rotates in the pond with only a part of it submerged in water. The pond has 40 cm depth, while the paddlewheel can go 25.5 cm deep in the water. The 4.5 cm of clearance from the bottom disturbs the uniformity of the flow. Additionally, the water is not always pushed horizontally by the paddlewheel. The VIV system shown in Fig. 1 (right) consists of a cylinder with 6 cm diameter, reels, reel beddings, springs and a frame that connects the VIV system to RWP. This system has been installed to pilot-scale RWP present at the roof of the Istanbul Technical University (ITU) Environmental Engineering Department.

All the experiments were carried out at a water level of 30 cm. The vertical amplitude and oscillation frequency of the cylinder was 6.5 cm and 1.24 s^−1^, as reported in [17].

### The PIV measurement system and software

**Fig. 2:**
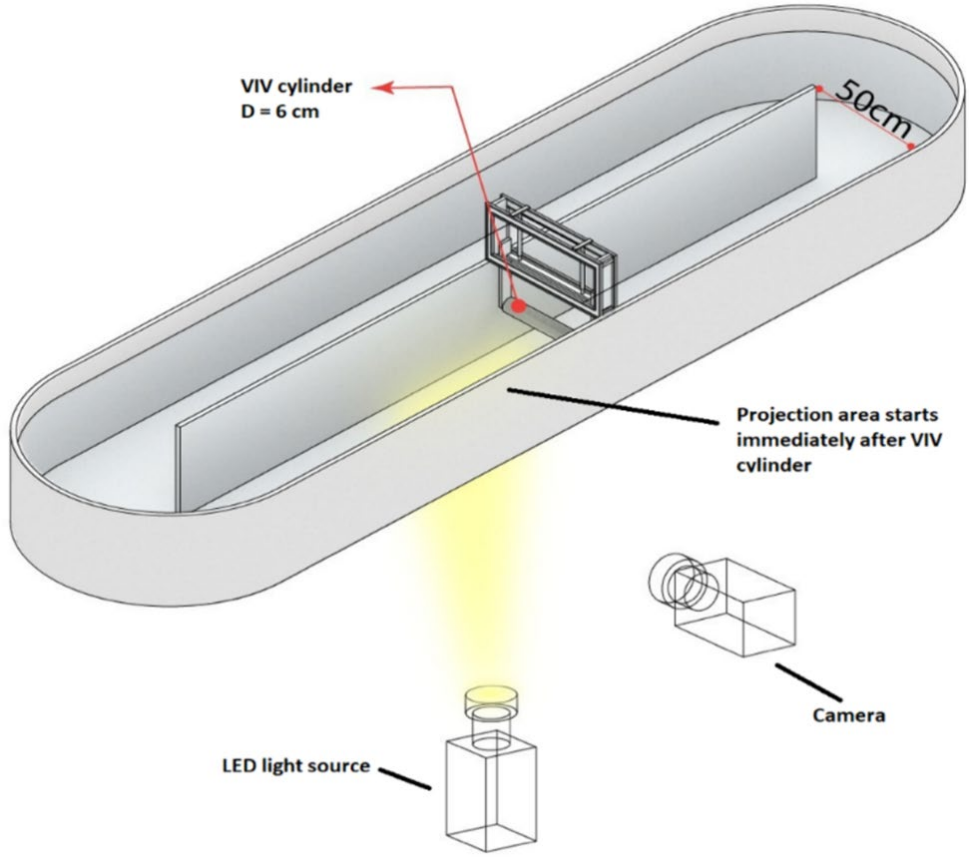
PIV system

The flow characteristics immediately downstream of the cylinder were visualized using the particle image velocimetry (PIV) technique. Light emitting diodes (LED) were used as the light source for flow measurement. The pond was filled with tap water to 30 cm head and seeded with polyamide particles having a mean 135 µm diameter. Parts of the wall and bottom of RWP were removed and replaced with glass. Light was introduced from below, as shown in Fig. 2. FlowSense USB 2 M-165 camera having a frame rate of 165 frames per second and 35 mm focal length was used to capture images. An LED illumination system equipped with a fiber light sheet optic was used as light source. The illumination system had 150 W power. All PIV hardware was provided by Dantec Dynamics Company. Data were processed using DynamicStudio software 6.8 and PIV-lab tool of MATLAB. Prior to processing, images were subjected to image contrast enhancement and background subtraction [18]. The projection area started at 1.5 m downstream of the paddlewheel, immediately after the VIV cylinder and covered 20 cm in the horizontal direction.

LED-based PIV systems are a cheaper alternative to laser pulsed systems, and have several other advantages including having a good luminous efficiency and very small spatial dimensions [19, 20]. On the other hand, it can be stated that their main disadvantages are the low coherence and high divergence of the emitted light [21] which may become a challenge for obtaining light sheets. To overcome this, a one-dimensional LED array is used with a fiber-optic illumination system and a cylindrical lens as described by Schmeling et al. [21].

### Calculation of cell trajectories

For calculation of cell trajectories, PIV data were exported to a spreadsheet. The spatial position of the particles at each time frame was calculated with the formula $D=u . t$, where D is the distance in the X and Y directions, $u$ is the velocity (recorded as cm/s) and $t$ is the time, 0.006 s. As the PIV camera captures 165 frames in 1 s, one time step corresponds to 0.006 s. The PIV projection area covers 20 cm in the horizontal direction and particles are tracked until they disappear at the end of the projection area. Three tracking positions are chosen with respect to pond depth. As pond depth is 30 cm, three equidistant positions—ports—tare chosen as *z* = 7.5 cm, *z* = 15 cm and *z* = 22.5 cm, where *z* = 0 is he pond bottom and *z* = 30 is the culture surface. A total of 75 particles were tracked from three ports.

**Fig. 3:**
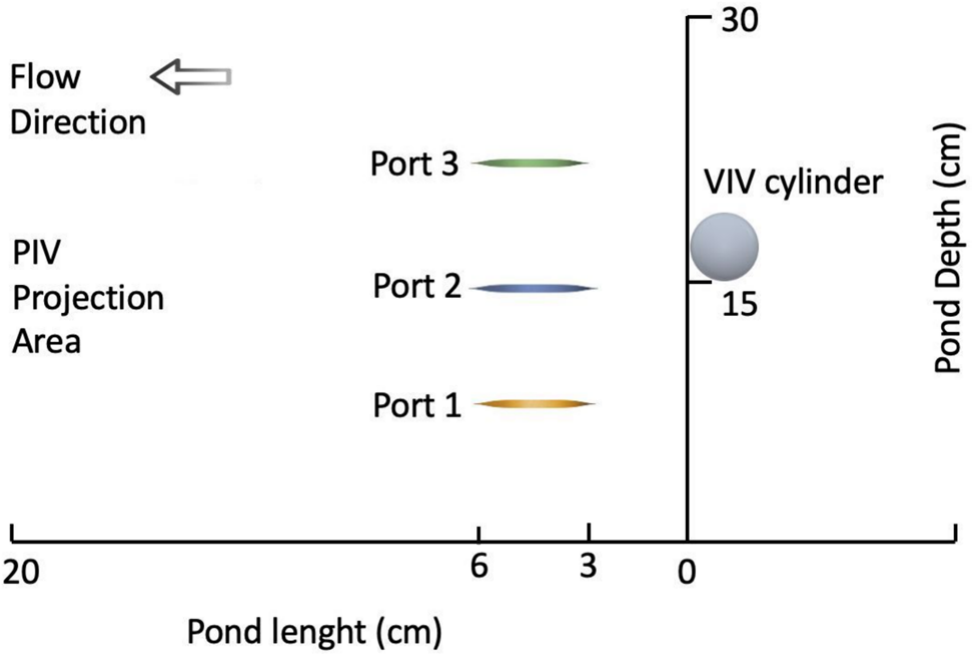
Initial positions of cells to be tracked

If the starting point of the projection area in the horizontal direction is considered *X* = 0, the initial positions of cells to be tracked are between *X* = 3 cm and *X* = 6 cm as shown in Fig. 3. Cells initially located between *X* = 0 and *X* = 3 cm were excluded. This was because of the vortex flow caused by cylinder oscillation; these cells moved in the -X direction and disappeared from the camera’s field of view. Enhancement on vertical mixing will be defined as the extent of cells’ vertical motion.

**Fig. 4:**
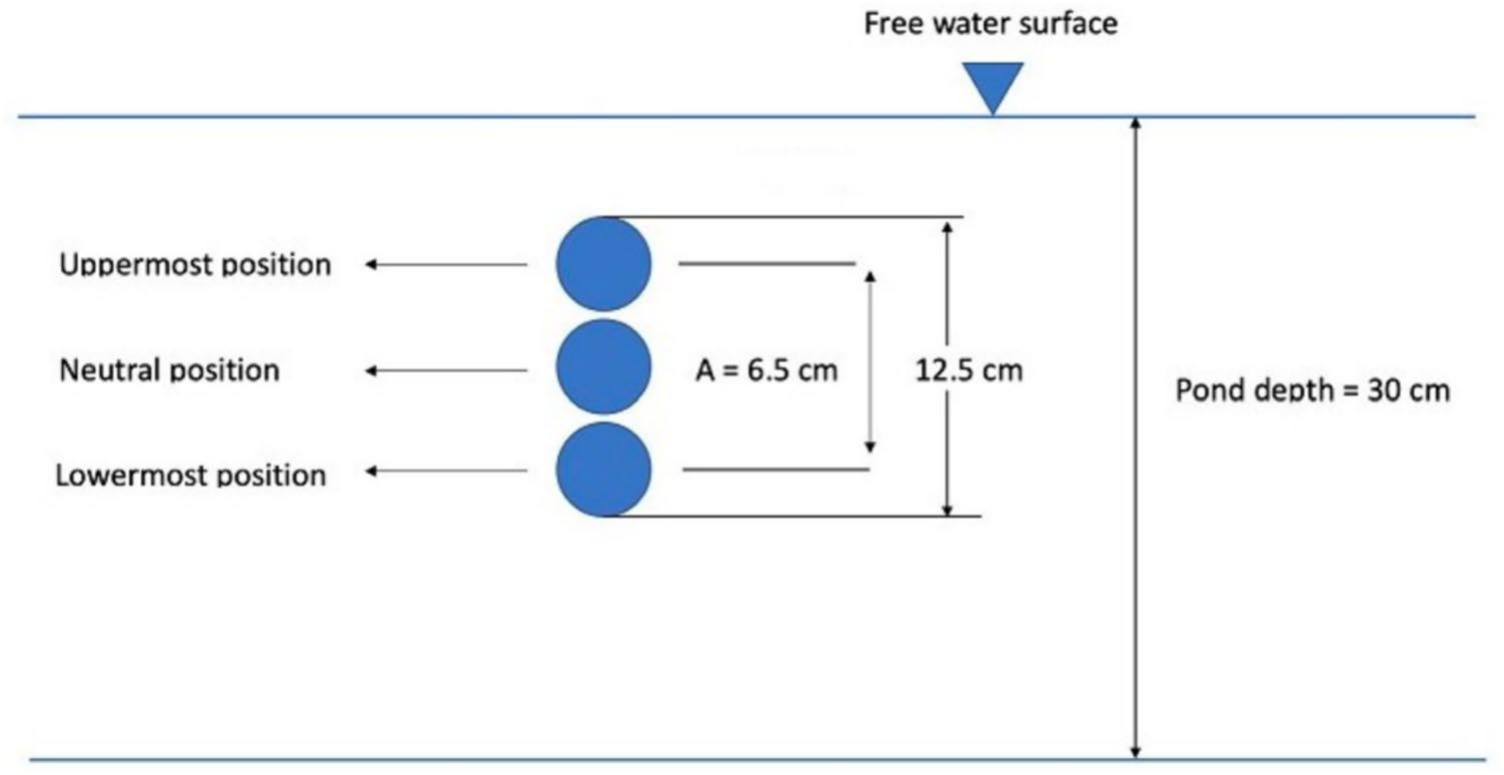
Positions of the cylinder during one oscillation (Figure not to scale) [17]

With the start of the flow, the VIV cylinder begins to oscillate. Initially, it is at the neutral position as depicted in Fig. 4.

At this juncture, the cylinder is positioned with its center 12 cm below the water surface, strategically chosen due to the observation of the highest oscillation amplitude at this location. Subsequently, the cylinder initiates its downward movement to reach the lowermost point, starts to move upward to attain the uppermost position, and returns to the neutral position, completing one full oscillation. To represent the average situation, the cylinder will be at the neutral position and moving downward when the trajectories start. The vertical amplitude and oscillation frequency of the cylinder were 6.5 cm and 1.24 s^−1^, as stated above.

### A new proposal for dividing the pond depth with respect to light availability

The light path through the depth of the reactors is typically categorized into two zones: light and dark. Algal cells dynamically move between these light and dark zones in response to fluid dynamic conditions during the cultivation period. It is widely acknowledged that the movement of cells between these two zones, often referred to as light/dark (L/D) cycles, significantly influences their growth.

The frequency of L/D cycles is known to affect the photosynthetic efficiency, as previous studies have repeatedly shown [22–25]. Furthermore, the light fraction, which is the duration of the light period in one L/D cycle, reportedly has a paramount effect on photosynthesis [25, 26].

Frequency of the L/D cycle, f_L/D_, is calculated as follows:

1 $$f_{{{\raise0.7ex\hbox{$L$} \!\mathord{\left/ {\vphantom {L D}}\right.\kern-0pt} \!\lower0.7ex\hbox{$D$}}}} = \frac{1}{{t_{c} }}$$

where light and dark periods of cells are represented as t_l_ and t_d_, respectively, while the total L/D cycle period is t_c_.

the light fraction ($\varnothing$) is defined as follows:

2 $$\emptyset = \frac{{t_{l} }}{{t_{c} }}$$

The averages of frequency and light fraction are calculated using Eqs. 3 and 4, respectively;

3 $$f_{{{\raise0.7ex\hbox{$L$} \!\mathord{\left/ {\vphantom {L D}}\right.\kern-0pt} \!\lower0.7ex\hbox{$D$}} \left( {av} \right) }} = \frac{{\mathop \sum \nolimits_{n = 1}^{n = 75} f_{{{\raise0.7ex\hbox{$L$} \!\mathord{\left/ {\vphantom {L D}}\right.\kern-0pt} \!\lower0.7ex\hbox{$D$}}}} }}{n}$$

4 $$\emptyset_{av} = \frac{{\sum\nolimits_{n = 1}^{n = 75} \emptyset }}{n}$$

In most of the studies focusing on the effect of L/D cycles, sharp light to dark transitions were considered. There are several approaches to determine the point for light to dark transition in the literature. Luo and Al-Dahhan [27] suggested that light intensity exceeding saturation should be defined as light zone, while some others suggested half of saturation intensity rather than saturation intensity [26].

Assuming that some parts of the reactor as “light” and “dark” has drawbacks and create confusion in fully evaluating the impact of light experienced by cells in real reactors, as cells are indeed exposed to a light gradient rather than direct light on/off pattern. Therefore, some part of the “dark” zone defined by Luo and Al-Dahhan [27] is indeed a light-limited zone, where net photosynthesis takes place. For this, we divide previously defined “dark” zone into two separate zones: namely light limited and dark. Thus, we propose three zones in a pond regarding light availability: (i) light-saturated zone, where light intensity is above saturation, (ii) light-limited zone where net photosynthesis takes place, but with its rate below the maximum and (iii) the dark zone where no photosynthesis takes place.

**Fig. 5:**
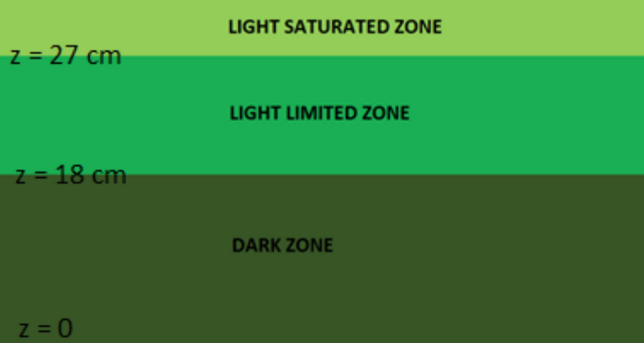
Proposed zones of the pond with respect to light availability

In this study, Luo and Al-Dahhan’s [27] approach was adopted to evaluate the characteristics of high-frequency L/D cycles, which considers the saturation intensity as the threshold value. As light distribution in pond varies with incident intensity and biomass concentration, the location of this threshold value is not constant and it is common to adopt certain approximations for the assessment of zones with respect to light availability. The location of transition between light saturation to light limitation was chosen as 3 cm below the culture surface as suggested by Chen et al. [16] to evaluate high-frequency L/D cycles in an RWP with 1 m total channel width and 2 m channel length. The culture depth was 30 cm, as in this study. About the light-limited zone of the pond where net photosynthesis takes place, Murphy et al. [14] reported that the photoactive part of the pond is first 12 cm from the culture surface. They carried out their experiments under conditions that simulate an RWP with 20 cm culture depth, measuring photosynthetic rates for gradually decreasing light intensities. Incident intensities in this study represented outdoor conditions. Light-limited part of the pond was assumed from *z* = 18 to *z* = 27 (where *z* = 0 is the pond bottom and *z* = 30 is the culture surface) as reported by Murphy et al. [14], and the zone where z is above 27 is assumed as light saturated, based on the approach of Chen et al. [16] (Fig. 5).

## Results and discussion

### Vertical distribution of flow velocity

The amplitude and frequency of VIV are closely related to the hydrodynamic behavior of the flow. To gain insights into how flow velocity varies with water depth, we first conducted velocity measurements at different depths in the pond without the VIV system, using two different paddlewheel rotation rates: 8 rpm and 12 rpm. Flow measurements were made using the PIV technique described under Section PIV measurement system and software.

**Fig. 6:**
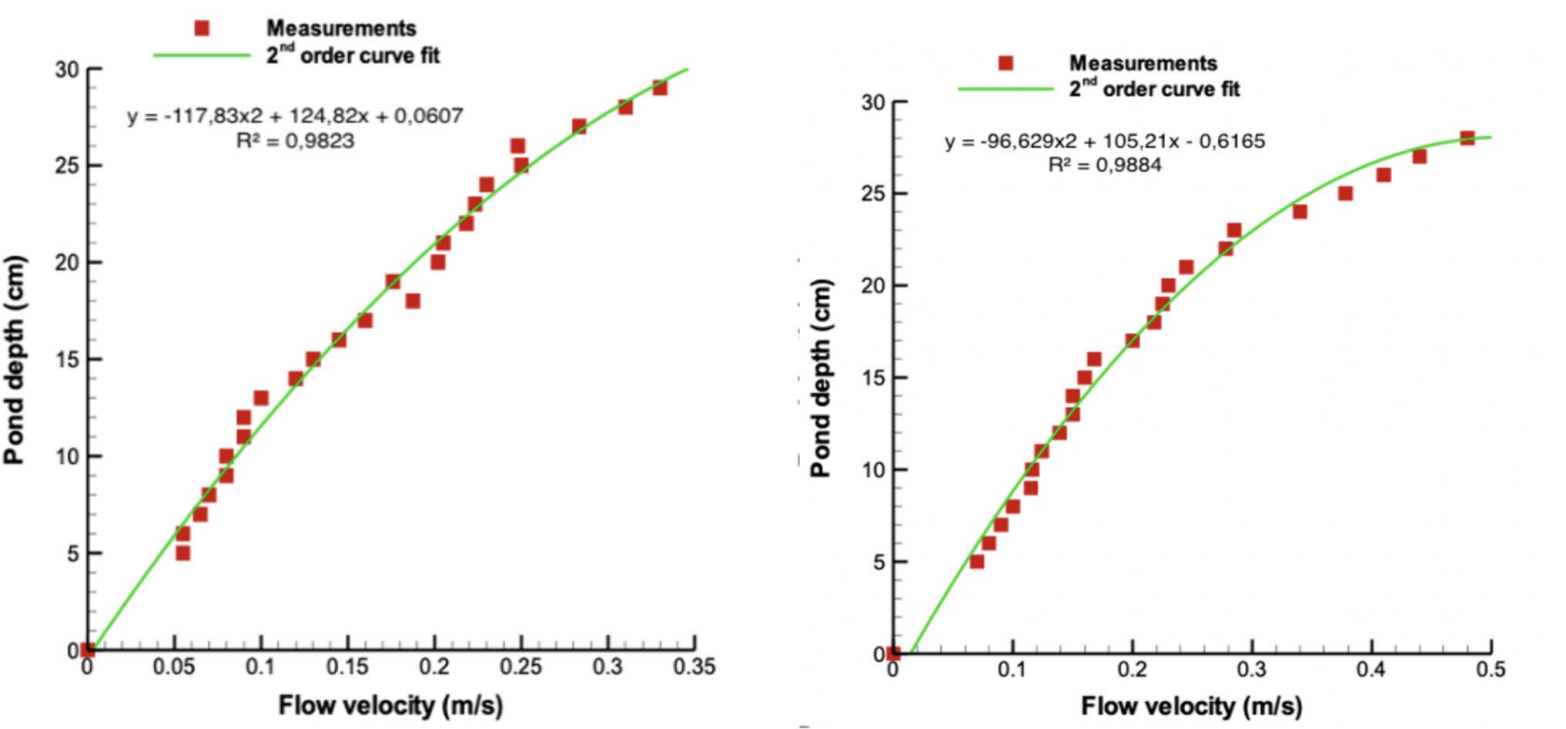
Vertical distribution of flow velocity through pond depth. 12 rpm paddlewheel speed (right), 8 rpm paddlewheel speed (left)

Our findings, presented in Fig. 6, demonstrate a velocity distribution that can be accurately modeled by a second-order polynomial. We also included fitted curves for both cases in the same figure for a better visualization of the results.

The lack of measurements in the first 5 cm can be justified by the existence of the boundary layer formed just above the bottom. Considering that the flow velocity is approximately ${V}_{5cm}\approx 0.06 m/s$ at 5 cm above the bottom for these two cases, we can make an approximation about the boundary layer thickness. The turbulent boundary layer thickness $\delta \left(x\right)$ is given by the equation:

5 $$\delta \left( x \right) \approx 0.37\frac{x}{{Re_{x}^{\frac{1}{5}} }}$$

where $x$ is the horizontal position of the measurement location and ${Re}_{x}$ is the Reynolds number of the flow. The Reynolds number is calculated by

6 $$Re_{x} = \frac{U \cdot x}{\vartheta }$$

Flow velocity measurements given in Fig. 6 are taken from $x=1.5 m$ away from the paddlewheel. In this case, the Reynolds number for the two cases is $Re_{x} = \frac{0.06 \cdot 1.5}{{10^{ - 6} }} = 90,000$. Substitution of this value into Eq. (5) gives a boundary layer thickness of approximately $\delta \left( x \right) \approx 5.6\,cm$. We know the velocity profile in the boundary layer region; therefore, we did not measure the flow velocities up to 5 cm above the bottom.

Cylinder oscillation with higher amplitudes and frequencies can be achieved through VIVs under uniform flow conditions, compared to non-uniform flow velocity distribution throughout the depth. On the other hand, it is challenging to achieve uniform flow conditions in an RWP. The rotation of the paddlewheel and the close proximity of the pond boundaries make it difficult to provide a suitable environment for uniform flow conditions.

Except for the case in which the paddle is perpendicular to the flow direction, there is always a vertical component of the flow. Thus, it is not reasonable to expect a uniform flow velocity with changing pond depth.

Due to non-uniform distribution of flow velocity through pond depth, the amplitude response of VIV is limited to 6.5 cm in pilot-scale RWP. To overcome this, the paddlewheel might be replaced with other mixing equipment. Several alternatives to paddlewheels are proposed in the literature such as propellers, Archimedes and centrifugal pumps and air lift pumps [9, 28, 29].

### Effect of VIV on flow in the RWP

Vertical amplitude obtained in the pilot-scale RWP was 6.5 cm, which means the cylinder covers 6.5 cm in the vertical direction during one oscillation with a frequency of 1.24 s^−1^, as stated above.

Cylinder oscillation happens as a result of pressure exerted by flow to the cylinder, which creates a radial force, while power available in the fluid is defined as force times velocity. Fluid power exerted on the cylinder can be calculated with the following formula:

7 $${P}_{fluid}=\frac{1}{2}\rho {U}^{3}DL$$

where P is the power available in the fluid, *ρ* is the fluid density, U is the flow velocity, D is the cylinder diameter and L is the cylinder length. With the vertical motion of the cylinder, the area swept by the cylinder becomes (D + 2A)L, where A is the oscillation amplitude. In this case, Eq. 7 can be rewritten as

8 $${P}_{fluid}=\frac{1}{2}\rho {U}^{3}\left(D+2A\right)L$$

**Fig. 7:**
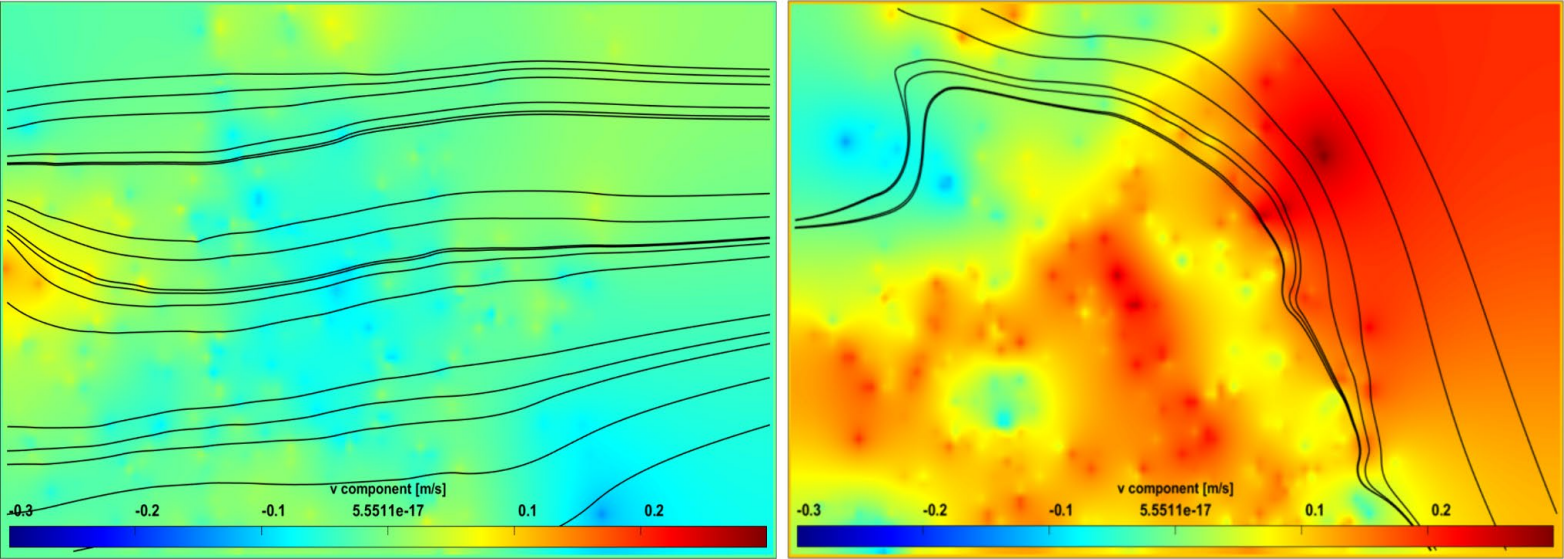
PIV visualizations of vertical flow velocities in the raceway pond. Conventional RWP (left), and RWP with the VIV system (right)

**Fig. 8:**
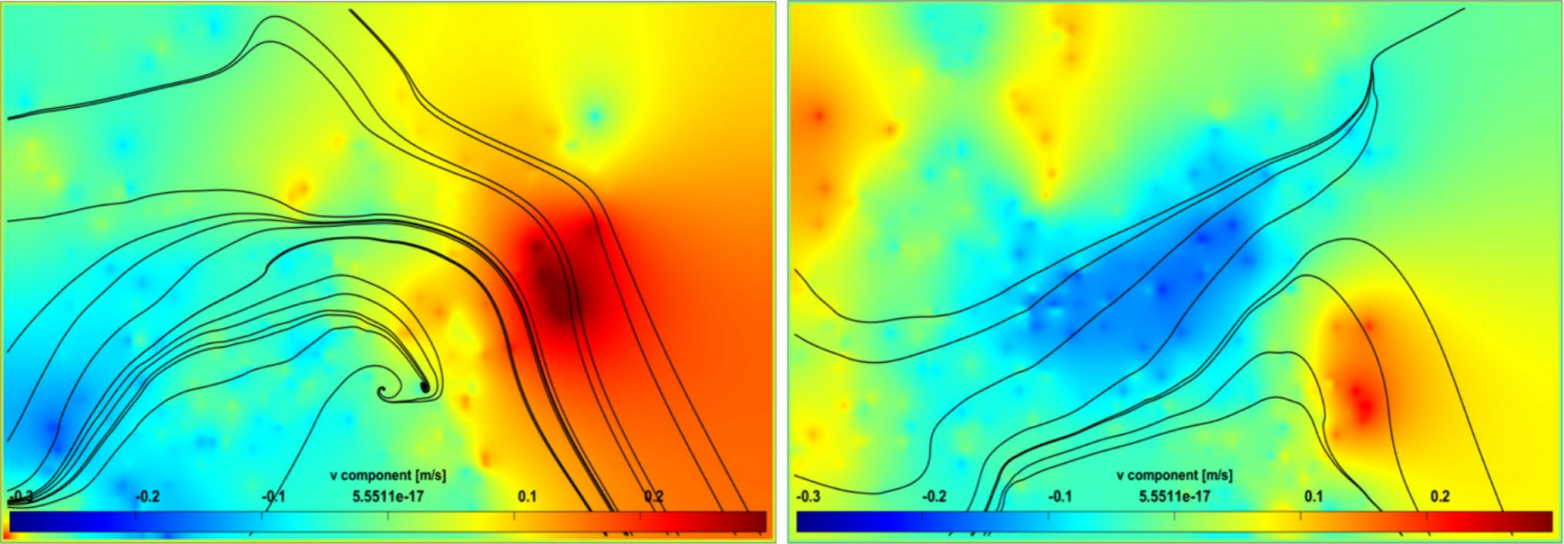
PIV visualizations of vertical flow velocities in the RWP with the VIV system. The cylinder is at neutral position and moving down (left) and lowermost position (right)

The projection region covers 20 cm in the horizontal direction immediately after the VIV cylinder and 15 cm in the vertical direction (from *z* = 15 to *z* = 30, where *z* = 0 is the pond bottom and *z* = 30 is the culture surface). The green color in the legend (Figs. 7, 8) represents the minimal vertical component of flow, which indicates that the flow is only in the horizontal direction, whereas red and blue colors represent strong vertical motion in upward and downward, respectively. Figures 7 (right) and 8 represent three different moments of the cylinder during one oscillation: from uppermost to neutral, neutral and lowermost, and moving downward. Owing to the nature of VIV motion, cells are displaced downstream toward the upper regions of the pond as the cylinder moves downward, and toward deeper sections when the cylinder moves upward, both movements occurring with equal magnitude. Streamlines in Fig. 7 (left) show that the flow almost moves in an orderly fashion and slight vertical flow is observed, when the VIV system is removed. This is in accordance with the literature as previous studies have repeatedly shown.

Figure 7 (right) shows the vertical velocity field captured for the case in which the VIV system was installed, when the cylinder was moving down, between the uppermost and neutral positions. The cylinder moves downward and vertical flow is observed in the + z direction at its downstream. As streamlines show, this motion sufficiently directs cells to the upper part: either light-limited or light-saturated zones. The cylinder is at its neutral position and moving down as shown in Fig. 8 (left). The flow created at the immediate downstream is toward the free surface. After a certain length (which is called the vortex formation length), the flow is evened or directed downward due to the vortex shedding phenomenon. In Fig. 8 (right), the cylinder is at its lowermost position. The vertical flow velocities are lower in this case as the vortices are suppressed due to the close bottom boundary. The bottom of the RWP weakens the vortex and therefore decreases the vertical flow velocities.

### Cell trajectories

The nature of vertical motion at the downstream of VIV is discussed in Section Effect of VIV on flow in the RWP. The effect of VIV motion in cells is directly related to oscillation amplitude of the cylinder. For the cells tracked from the first port (Fig. 3 and Table 1), the effect of VIV could not be observed due to relatively low amplitude. Cells released from this port exhibited a horizontal motion and stayed in the dark zone throughout the flow visualization.

**Table 1:** - Positions of ports where cells were tracked from

Port	Position	Zone
1	7.5 cm above the pond bottom	Dark
2	15 cm above the pond bottom	Dark
3	22.5 cm above the pond bottom	Light limited

**Fig. 9:**
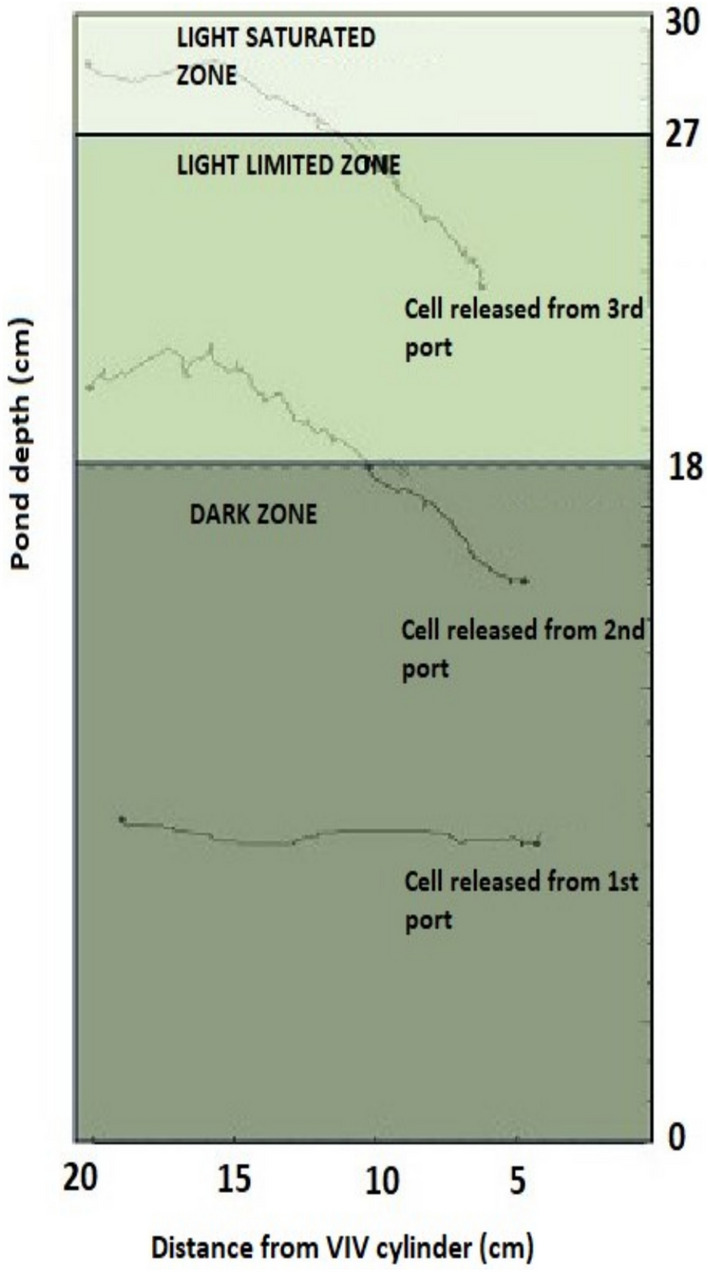
Cell trajectories from the first port (down), second port (middle) and third port (up). Particle paths indicate strong vertical mixing in RWP

On the other hand, it has been observed that the cells tracked from the second and third ports effectively entered the light-limited and light-saturated zones of the pond, respectively. Examples of cell trajectories are shown in Fig. 9.

**Fig. 10:**
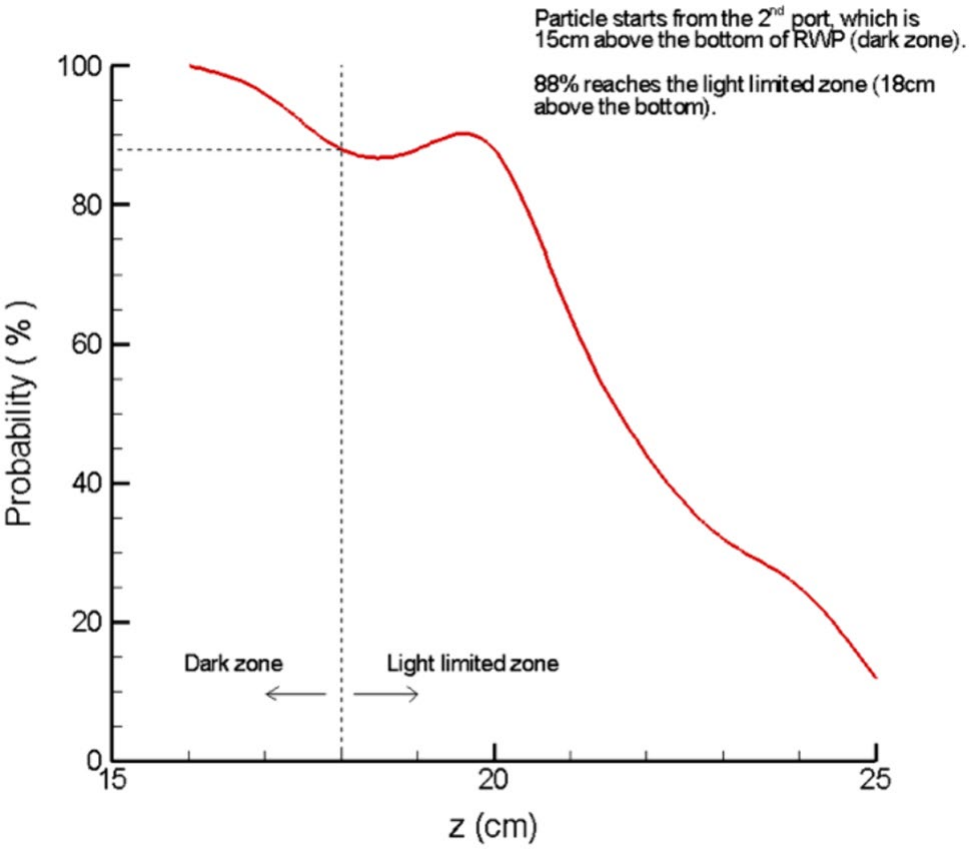
Probability distribution of the final vertical positions of cells released form the second port

On average, about 44% of the cells initially in the dark zone (first and second ports) reached the light-limited zone, while 88% of the cells released from the second port reached the light-limited zone from the dark zone. 100% of cells released from third port, which is in the light-limited zone, reached the light-saturated zone. Probability distribution showing the final vertical positions of cells released from the second port is shown in Fig. 10.

Cells released from the third port were already in the light-limited zone, as stated above. However, as the light intensity in the pond increases sharply, light availability and photosynthetic rates of these cells would be greatly improved as these cells move toward the culture surface.

**Fig. 11:**
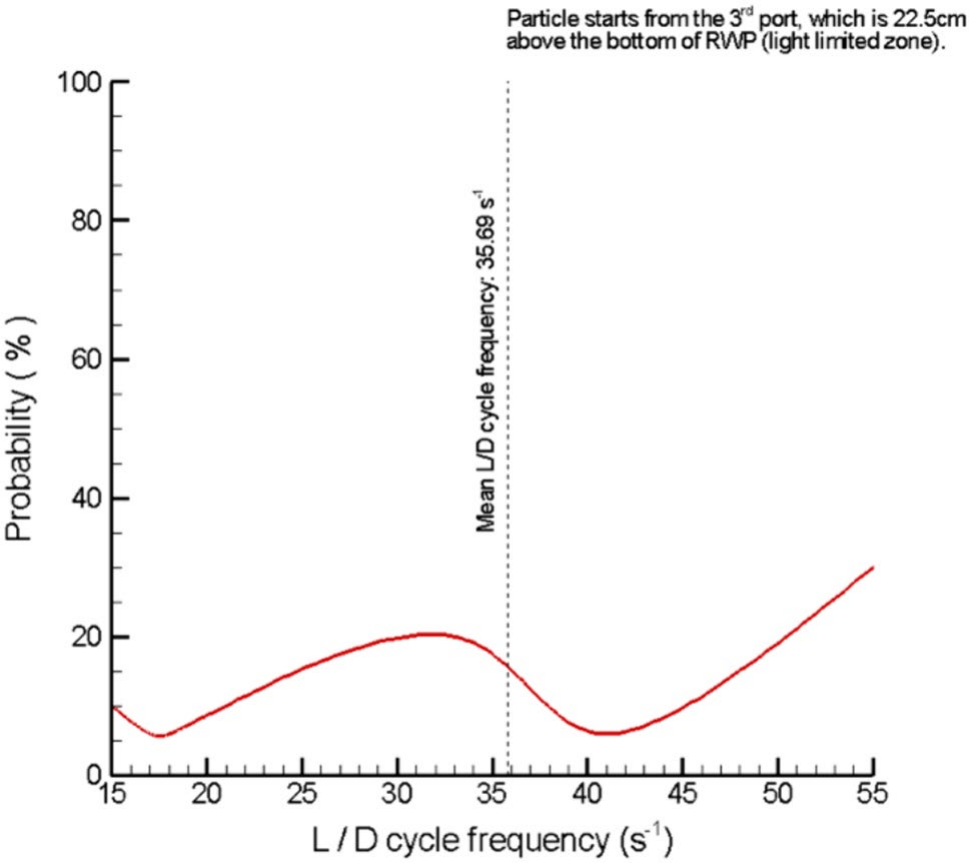
Probability distribution of L/D cycle frequencies experienced by cells released from the third port

With the VIV motion, 33% of all the tracked cells—100% of cells released from the third port—are exposed to high-frequency L/D cycles, moving between light-limited and light-saturated zones. The average frequency was 35.69 s^−1^. Light fractions ranged from 0.09 to 0.91, with an average of 0.49. Probability distribution of L/D cycle frequencies experienced by cells released from the third port are given in Fig. 11.

For a comprehensive assessment of the influence of VIV on cellular motions, changes in cell transitions between light-limited and dark zones were thoroughly examined. The oscillation of the cylinder induces cell movement between the light-limited and dark zones, akin to the transitions observed between the light-saturated and light-limited zones which was examined to investigate the nature of high-frequency L/D cycles. 84% of the cells released from the second port underwent cycles between the light-limited and dark zones, exhibiting an average frequency of 55 s⁻^1^. The average light fraction for these cells was calculated as 0.67.

High-frequency L/D cycles are recognized for their positive impact on biomass productivity compared to continuous illumination due to the so-called flashing light effect. On the other hand, achieving high-frequency L/D cycles in RWPs proves challenging due to restricted turbulence and vertical mixing. Conversely, medium-frequency cycles are recognized for their minimal impact on biomass productivity in this context [30]. Decreased productivities are reported for L/D cycle frequencies ranging from 0.1 to 0.01 s^−1^ when compared to continuous illumination [25, 31, 32]. Furthermore, light fraction, which is defined as the ratio of time cells spend in the light zone to total cycle time, is reported as another important parameter that influences light utilization efficiency [25, 26, 32]. Brindley, et al. [25] observed consistent decrease in photosynthetic rate when the light fraction gradually decreased from 0.2 to 0.05 for L/D cycles with 1 s^−1^ frequency for their study conducted in a flat panel photobioreactor. For much higher L/D cycle frequency of 50 s^−1^, this pattern was still applicable, as the photosynthetic rate decreased more than 2.5-fold when the light fraction was decreased from 0.2 to 0.05. For medium-frequency L/D cycles (0.1 s^−1^), the same authors reported a decrease of 3.85-foldsin biomass productivity, when the light fraction decreased tenfold, from 0.5 to 0.05. L/D cycle frequencies in this work were found to be within a range of 15–55 s^−1^, which is reported to be suitable for increased light utilization efficiency [25].

It is important to acknowledge that, although the frequency of L/D cycles is promising, only 33% of cells (only the cells released from the third port) were observed to experience this phenomenon. In addition, the transfer of cells from dark to light-limited zone (cells released from the second port) had an impact on overall light availability. In a light-limited environment, the rate of photosynthesis is directly proportional to light intensity. As light penetrates the culture depth, it undergoes an exponential decrease. Consequently, cells moving from dark to light-limited zone experience an increase in the rate of photosynthesis proportional to the available light. Utilizing VIVs in RWPs which directs cells toward zones that offer improved light availability enhances photosynthetic efficiency and, in turn, overall biomass production. Therefore, it is believed that approximately a 20% increase in biomass production reported in our previous study [17] resulted from the combined effects of increased L/D cycle and improved light availability. On the other hand, cells released from the first port, which was positioned very close to the pond bottom, exhibited minimal vertical movement, persisting in the dark zone due to the restricted influence of VIV oscillation in this area. Enhancing the oscillation amplitude to effectively direct these cells toward the light-limited zone of the pond holds the potential to further optimize biomass production in VIV-implemented RWPs.

### Further improvement of vertical mixing by VIVs

The oscillation amplitude of the VIV cylinder inside the pond is limited due to non-uniform flow conditions, as explained in detail in Section Vertical distribution of flow velocity.

**Fig. 12:**
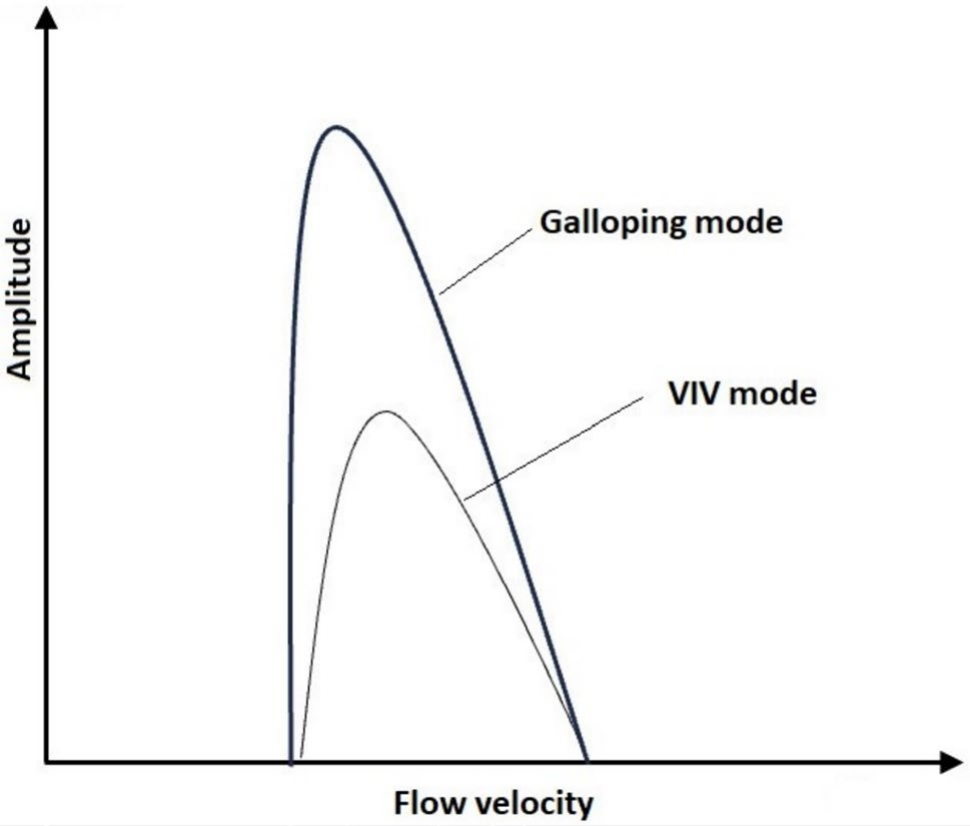
VIV and galloping modes

To achieve further improved oscillation amplitude and therefore vertical mixing, the VIV system must be transitioned to galloping mode (Fig. 12) where the cylinder oscillation is unstable and very high amplitudes (and frequencies) can be achieved [33].

Flow velocity and amplitude can be transformed into non-dimensional form with the following equations:

9 $${U}^{*}=\frac{U}{{f}_{nw}D}$$

where U is the flow velocity, f_nw_ is the natural frequency in still water, which is 0.99, and D is the cylinder diameter. U* is non-dimensional flow velocity. Non-dimensional amplitude (A*) can be calculated with the following equation:

10 $${A}^{*}=\frac{A}{D}$$

U* and A* in this study was 5 and 0.54, respectively. For the U* values similar to this study, higher A* values were reported. Pan et al. [34] observed analogous A* responses at U* values close to 5, while Khalak and Williamson [35], as well as Wanderley et al. [36], achieved A* = 1 at the same U*. In contrast, Duranay and Kinaci [37] observed A* values exceeding 1 at U* = 3.

**Fig. 13:**
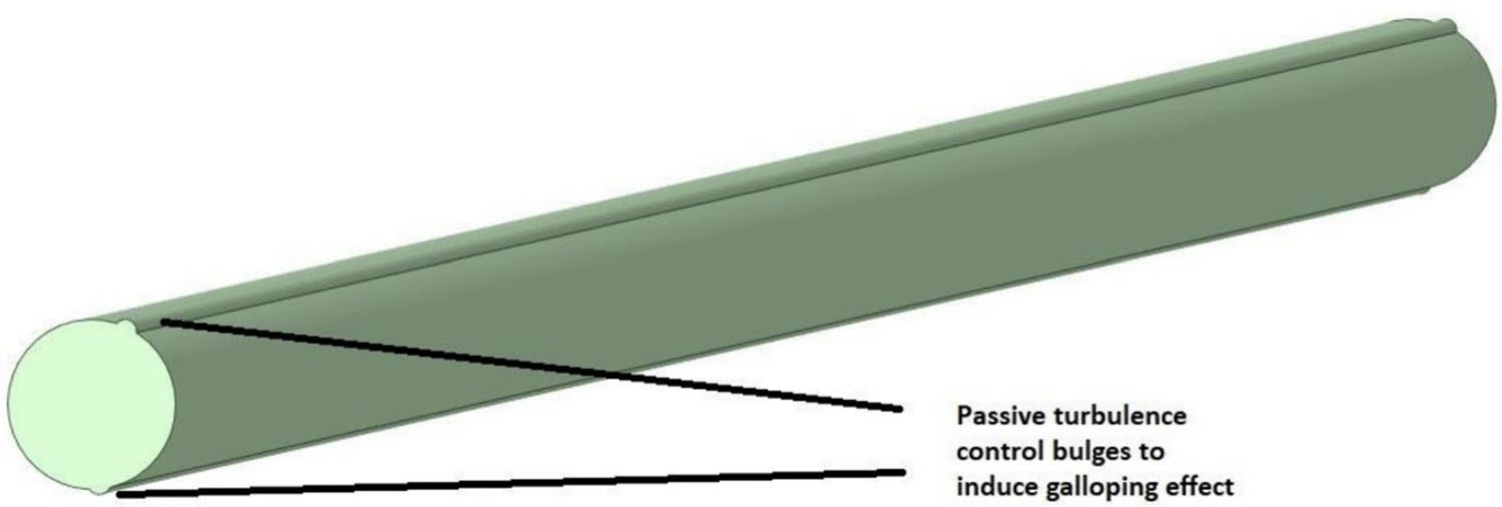
An example for passive turbulence control bulges implemented in the VIV cylinder

One of the most straightforward ways to induce galloping is by introducing passive turbulence control bulges, such as strips on the cylinder to initiate earlier boundary layer separation [38] as shown in Fig. 13.

This is expected to facilitate more significant improvements in vertical mixing, enabling better performance of the VIV system.

**Fig. 14:**
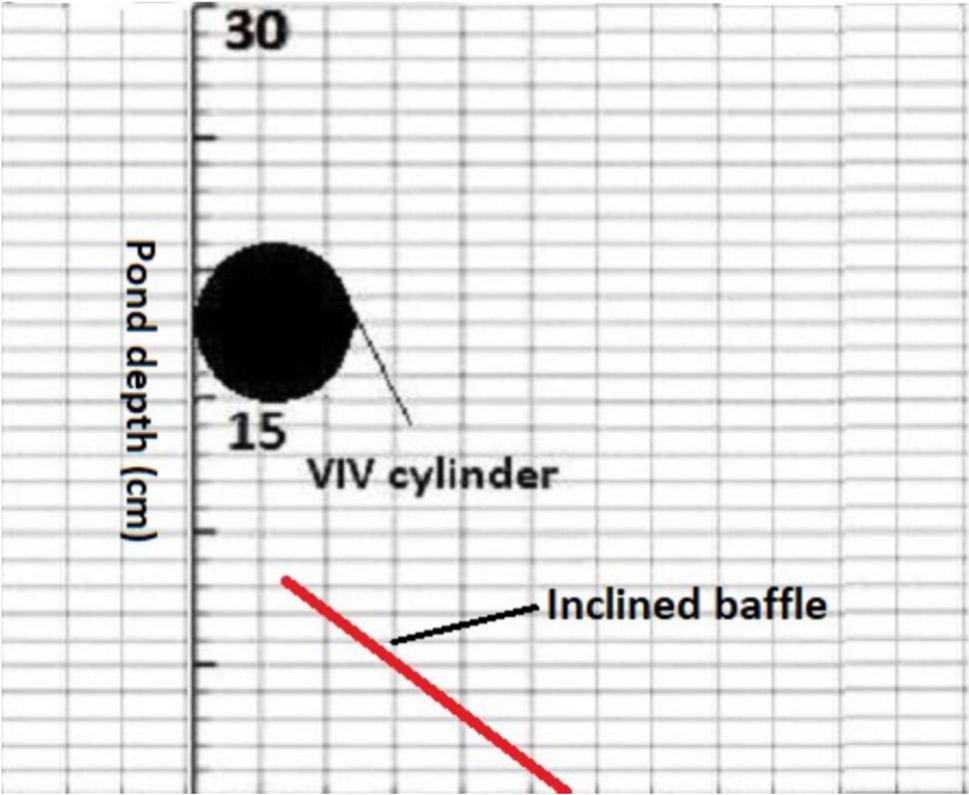
Example of the proposed inclined baffle

Another strategy for maximizing the effect of VIVs to enhance biomass production in RWPs involves integrating them with static baffles as described in Fig. 14. The concept involves the installation of static inclined baffles in the dark zone of the pond, preceding the VIV cylinder.

This strategic placement could facilitate the redirection of cells in the lower parts of the pond where the effect of VIV cylinder oscillation is not pronounced, to zones where it is effective. Subsequently, the VIV cylinder could carry these cells into the light-limited zone, enhancing their exposure to light for enhanced biomass production.

## Conclusions

In this study, fluid dynamic characterization of VIV-assisted RWP for improved vertical mixing was carried out. Cells were tracked to assess the effect of VIV on the vertical motion of cells which would otherwise move in the horizontal direction using the flow visualization technique. For this, the pond depth was hypothetically divided into three zones with respect to light availability, namely, light-saturated, light-limited and dark zones. The following conclusions were drawn from the study:

1. Dividing pond depth into three zones, namely, dark, light-limited and light-saturated zones, is found useful for understanding improvement in light availability resulting from VIV cylinder oscillation.
2. VIV cylinder oscillation can effectively transfer cells from light-limited to light-saturated zone. However, only a part of the cells were transferred from dark to light-limited zone and none to the light-saturated zone.
3. L/D cycle frequencies achieved by VIV cylinder oscillation is significantly higher than those reported for conventional RWPs, enabling increased photosynthetic efficiency.
4. It is believed that approximately a 20% increase in biomass production reported in our previous study resulted from the combined effects of increased L/D cycles and improved light availability, resulting from directing cells from dark to light-limited zone.
5. The effect of VIV cylinder oscillation could not be observed for the cells residing in the lower parts of the pond due to limited oscillation amplitude achieved in established flow conditions which are common in full-scale RWPs.
6. To enhance the impact of VIV to cover whole channel depth, two strategies can be implemented: (i) amplifying the oscillation amplitude of the VIV cylinder by inducing galloping effects, and (ii) directing cells in the deeper parts of the channel toward zones where the effect of VIV cylinder oscillation is observed through the use of static structures, such as inclined baffles.

## Data Availability

No datasets were generated or analysed during the current study.
